# Immunohistochemical Analysis of Lymphocyte Subpopulation in Peri-implant Peripheral Giant Cell Granuloma

**DOI:** 10.5152/eurasianjmed.2026.261687

**Published:** 2026-10-19

**Authors:** Alparslan Dilsiz, Rabia Demirtaş, Ayşegül Türksoy, Ayşegül Tanas, Fevziye Bektaş

**Affiliations:** 1 Department of Periodontology, Atatürk University Faculty of Dentistry, Erzurum, Türkiye; 2 Department of Pathology, Atatürk University Faculty of Medicine, Erzurum, Türkiye

**Keywords:** CD3, CD4, CD8, CD20, CD79a, dental implants, gingival overgrowth, gingivectomy, lymphocyte subpopulation, peripheral giant cell granuloma, periodontal debridement

## Abstract

**Background:**

Peripheral giant cell granuloma (PGCG) is a benign inflammatory lesion affecting the gingival tissues of the oral cavity and is among the more frequently encountered reactive lesions. This study aimed to characterize the distribution of lymphocyte subpopulations (CD3, CD4, CD8, CD20, and CD79a) in peri-implant PGCG and to compare their expression profiles with those observed in normal gingival tissues.

**Methods:**

Twenty-eight systemically healthy, non-smoking individuals diagnosed with peri-implant PGCG were enrolled in the study. Tissue specimens from peri-implant PGCG lesions were obtained during surgical excision and assigned to the test group, whereas biopsies of adjacent clinically normal gingiva served as the control group. Immunohistochemical staining was performed to evaluate the distribution of the selected lymphocyte subpopulations.

**Results:**

All tissue samples from both the test and control groups showed positive staining for CD3, CD4, CD8, CD20, and CD79a. The proportion of CD4-positive cells was significantly greater in peri-implant PGCG specimens (53.5%) than in normal gingival tissues (20%) (*P* < .05). In contrast, CD20-positive cells were significantly less abundant in the test group (10.5%) compared with the control group (30%) (<.05).

**Conclusion:**

Immunohistochemical assessment of lymphocyte subpopulations represents a well-recognized approach for defining the immune characteristics of inflammatory lesions. The present findings demonstrated that both T- and B-lymphocytes are present in peri-implant PGCG and normal gingiva; however, their relative distributions differ significantly between the 2 tissues. These differences may reflect distinct immunological mechanisms associated with normal and diseased gingival tissues.

**Main Points**

- Peripheral giant cell granuloma (PGCG) of the oral cavity is a relatively common non-neoplastic inflammatory lesion of gingival tissues.
- The study provides information about peri-implant PGCG. The research can give an idea for the immunopathology of peri-implant PGCG.
- Immunohistochemical analysis of oral soft tissues is central to the differential diagnosis of gingival overgrowths and a practical method for characterizing immunological profiles in infectious inflammatory gingival overgrowth diseases.
- T and B cells are present in peri-implant infectious and normal gingiva. There is a distinct immunopathology of normal versus peri-implant PGCG.

## Introduction

Peripheral giant cell granuloma (PGCG) is an uncommon benign lesion affecting the gingival tissues of the oral cavity. Although its exact pathogenesis remains unclear, local irritative factors, particularly trauma and infection, are considered to be the main contributors to its development.^1^^-^^4^ Peripheral giant cell granuloma is generally regarded as a reactive inflammatory lesion, and hormonal factors have also been proposed to contribute to its development. Clinically, it usually presents as a painless, asymptomatic gingival enlargement involving either the maxilla or the mandible. Epidemiological studies have reported a slight female predominance, with the mandibular region being affected more frequently than the maxilla. Histopathologically, PGCG is characterized by the presence of numerous multinucleated giant cells and is thought to arise from cells derived from the periodontal ligament or periosteum.^2^^,^^3^ Although conventional PGCG and its peri-implant counterpart exhibit similar histopathological characteristics, the mechanisms underlying their development remain incompletely understood. The etiology of peri-implant PGCG has yet to be fully elucidated; however, several local factors, including inadequate oral hygiene, poorly fitting prosthetic restorations, occlusal conditions, a previous history of periodontal disease, persistent inflammation, and corrosion of dental implants, have been suggested as potential predisposing factors. Surgical excision, with or without peripheral osteotomy and curettage of the underlying bone, remains the standard therapeutic approach to achieve complete lesion removal and reduce the likelihood of recurrence.

The adaptive immune response is mediated through both humoral and cellular mechanisms, in which lymphocytes represent the principal effector cells. T and B lymphocytes are found in both normal and inflamed gingival tissues and contribute substantially to the immunological processes associated with periodontal disease. Periodontal diseases are initiated by bacterial biofilms whose microbial products stimulate gingival epithelial cells to produce pro-inflammatory cytokines and increase the expression of adhesion molecules, thereby facilitating leukocyte migration into the affected tissues. Ongoing bacterial stimulation subsequently promotes the accumulation of inflammatory infiltrates composed predominantly of T lymphocytes and macrophages. In addition to orchestrating cell-mediated immune responses, T lymphocytes regulate antigen-specific antibody production and polyclonal activation of B cells. Previous studies have suggested that T cells are the predominant lymphocyte population in gingivitis, whereas B-cell infiltration becomes more pronounced during periodontitis through T-cell-dependent regulatory mechanisms.^5^^-^^29^ Published studies have reported inconsistent findings regarding the relative distribution of lymphocyte subpopulations in gingival tissues and their association with periodontal health.^30^^,^^31^ Although lymphocyte subpopulations have been extensively investigated in periodontal tissues and biological fluids, no research has yet compared these subpopulations between peri-implant PGCG and normal tissues.^5^^-^^30^ To the best of the authors’ knowledge, no studies have been published concerning directly comparing the immunohistochemical profiles of lymphocyte subpopulations in peri-implant PGCG and normal gingival tissues. Accordingly, this study was designed to characterize CD3-, CD4-, CD8-, CD20-, and CD79a-positive lymphocyte subpopulations and compare their distribution between the 2 tissue types.

## Material and Methods

This prospective observational paired tissue study received approval from the Clinical Research Ethics Committee of University of Atatürk (AU-IIREB:239; April 17, 2026) and was performed in accordance with the ethical principles of the Declaration of Helsinki for research involving human participants. Prior to enrollment, written informed consent was obtained from all participants in compliance with the 1975 Declaration of Helsinki, as revised in 2013. The study protocol was registered at ClinicalTrials.gov (NCT07551310). Participant recruitment and clinical procedures were carried out between April 18 and July 12, 2026. This study used no artificial intelligence (AI) or large language model (LLM)-based tools during the preparation of this manuscript.

### Sample Size

The required sample size was determined using G*Power software based on a 2-tailed independent-samples *t*-test. The calculation was performed assuming a significance level (*α*) of 0.05, a statistical power of 80%, an allocation ratio of 1 : 1, and a large effect size (Cohen’s *d* = 0.8). Based on these parameters, the minimum required sample size was calculated as 26 participants for each group. The analysis yielded 50 degrees of freedom, a critical *t*-value of 2.01, and an achieved statistical power of 0.81.^32^ The total sample size was 26 and was rounded up to 28 to compensate for participant losses.

### Selection of Patient

The study was conducted on a total of 211 patients (peri-implant biopsies), of which 35 (16.6%) were diagnosed as peri-implant PGCG presenting to Atatürk University. Participants were eligible if they met the following criteria: (1) no systemic disease or relevant medical condition, (2) no history of medications known to induce gingival overgrowth, and (3) non-smoker. Seven patients who did not meet the inclusion criteria for the study were excluded.

### Specimen Collection

Biopsy specimens from peri-implant PGCG lesions were obtained at the time of surgical excision and constituted the test group, whereas clinically normal gingival tissue adjacent to the lesion was collected as the control group. Gingivectomy and gingivoplasty procedures were performed under local anesthesia using conventional surgical blades. To preserve tissue architecture, local anesthetic solution was not injected directly into the biopsy site, and care was taken to avoid compression or distortion of the specimens during tissue harvesting. Each excised lesion was divided into 2 portions, 1 of which was submitted to the Department of Pathology for routine histopathological examination.

Immediately after collection, all biopsy specimens were immersed in 10% neutral buffered formalin and fixed for 48 hours. Tissue processing was subsequently carried out for 14 hours using a Thermo Fisher Scientific Shandon Excelsior ET tissue processor. The processed specimens were embedded in paraffin, sectioned at a thickness of 4 μm, and stained with hematoxylin and eosin. Morphological evaluation of the prepared slides was independently performed by 2 pathologists using a Zeiss Scope A1 light microscope.

### Immunohistochemical Study

Four-micron sections were cut from the tissue blocks, and the slides containing the tissues were placed in a Roche Ventana automated immunohistochemistry staining system (Ventana Roche, USA) after being left in a 70°C drying oven for 15 minutes. The tissues in the instrument were sequentially deparaffinized and dehydrated, then treated with ULTRA Cell Conditioning Solution, hydrogen peroxide, and CD3, CD4, CD8, CD79a, and CD20 antibodies. Immunohistochemical staining for CD3, CD4, CD8, CD20, and CD79a was independently evaluated by 2 pathologists. For each case, the area with the highest density of inflammatory cells was selected, and positive cells were manually counted in a single microscopic field at ×400 magnification. The percentages of immunopositive cells were calculated based on the total number of cells evaluated in the selected field. No significant difference was observed between the assessments of the 2 pathologists. In cases of disagreement, the slides were jointly re-evaluated and a consensus was reached.

### Statistical Analysis

Data analysis was conducted using SPSS 20.0 statistical software (SPSS Inc.Chicago, IL, USA). To compare the proportions of CD3, CD4, CD8, CD20, and CD79a cells between the test and control groups, the Wilcoxon signed-rank test was utilized. Statistical significance was established at a level of *P* < .05.

## Results

All surgical procedures and subsequent healing phases for the study participants were completed without complications. Clinical and demographic data for the 2 groups are presented in Table 1. Peri-implant PGCG was found to predominantly affect females (64%) (18 females, 10 males), with ages ranging from 39 to 66 years (mean age: 52.6 years), and occurred mostly in the mandible (68%). In most instances (68%), the lesion diameter was less than 15 mm. Histological evaluations revealed typical features, including the proliferation of fibrohistiocytic cells and numerous multinucleated giant cells within a vascularized stroma (Figure 1).

CD3, CD4, CD8, CD20, and CD79a cells were identified in all specimens from both groups (Figures 2-6). Significant differences in lymphocyte subpopulations proportions were found between the test and

control groups (Table 2). The proportion of CD4 cells was significantly higher (*P* < .05) in the test group (53.5%) compared to the control group (20%). Conversely, the proportion of CD20 cells was significantly lower (*P* < .05) in the test group (10.5%) than in the control group (30%). The mean CD4/CD8 ratio was also higher in the test group (53.5/12.5) compared to the control group (20/13.5).

## Discussion

This study evaluated and compared the expression of CD3, CD4, CD8, CD20, and CD79a in peri-implant PGCG versus normal gingival tissue. To date, this is the first study to analyze these specific lymphocyte subpopulations in peri-implant PGCG. The distribution of lymphocyte subpopulations in peri-implant PGCG was identified as: helper T cells (CD4) > pan-T cells (CD3) > suppressor T cells (CD8) > plasma cells (CD79a) > B cells (CD20).

Peripheral giant cell granuloma is categorized as one of several non-neoplastic inflammatory lesions, alongside pyogenic granuloma (PG), irritation fibroma, and others. Clinically, it presents as a soft, reddish-blue nodular mass, with diagnosis relying on clinical and histopathological findings. It closely resembles PG and is characterized histologically by mesenchymal cell proliferation, multinucleated giant cells, and high vascularity.^1^^-^^3^ Histopathological analysis is essential to distinguish PGCG from other oral lesions, which is why it was used for diagnostic accuracy in this study.

Lymphocytes are critical to both humoral and cell-mediated immunity, playing a central role in directing non-specific inflammatory responses and immunoregulation.^7^^-^^10^ In diseased periodontal tissues, a mononuclear inflammatory infiltrate containing T cells, B cells, and plasma cells is present. The study investigated these subpopulations in peri-implant PGCG using periodontal tissue biopsies. The findings showed that both T and B cells were present in both groups but in varying proportions, aligning with previous research.^33^^-^^35^ While some studies suggest T cells dominate in gingivitis and B cells in periodontitis, others have reported different findings, such as higher B-cell counts in aggressive periodontitis.^6^^,^^8^^,^^12^^,^^16^^,^^20^^,^^21^^,^^23^^,^^29^

In this study, CD4 T cells were more numerous in peri-implant PGCG than in normal tissue, while CD20 B cells were less frequent. CD79a-positive plasma cells, which appear early in B-cell maturation, were also detected. The number of CD79a cells was not significantly lower in the test group and were found scattered or near T and B cells. The CD4/CD8 ratio is an important indicator of immune function.^10^^,^^11^ While some reports suggest this ratio remains constant between normal and diseased tissues, others have found it to be lower or higher in diseased states. Meng et al show that this ratio is the same in diseased and normal periodontal tissues.^25^^-^^29^ The results showed a higher CD4/CD8 ratio in peri-implant PGCG. This may be due to continuous local irritation from the trauma, leading to increased vascularity and immune activation.

Few cases of peri-implant PGCG have been documented. Systematic reviews show a small percentage of PGCG lesions are associated with dental implants.^3^^,^^33^^-^^35^ The findings confirm that peri-implant PGCG is more common in adult females and typically occurs in the posterior mandible.^1^^-^^3^^,^^33^^-^^35^ While some studies have compared markers like CD68 and CD34 in giant cell lesions, data on T and B cells in peri-implant PGCG remain limited. This study observed a predominance of CD4 T cells and a low number of CD20 B cells. T cells were distributed diffusely, likely participating in antigen recognition and macrophage activation.

The fact that control samples were obtained from clinically normal gingiva adjacent to the PGCG lesion could be considered a limitation of the present study.

In conclusion, within the limitation of the present clinical trial, analyzing lymphocyte subpopulations in tissues is an established method for characterizing immunological profiles in infectious diseases. This study demonstrated that while both T and B cells are present in peri-implant PGCG and normal gingiva, their proportions differ significantly, which reflects the distinct immunopathology of normal versus diseased gingival tissues. Further immunohistochemical studies should be conducted to determine the role of T and B lymphocytes in affecting the pathobiology of peri-implant PGCGs.

## Figures and Tables

**Figure 1.**
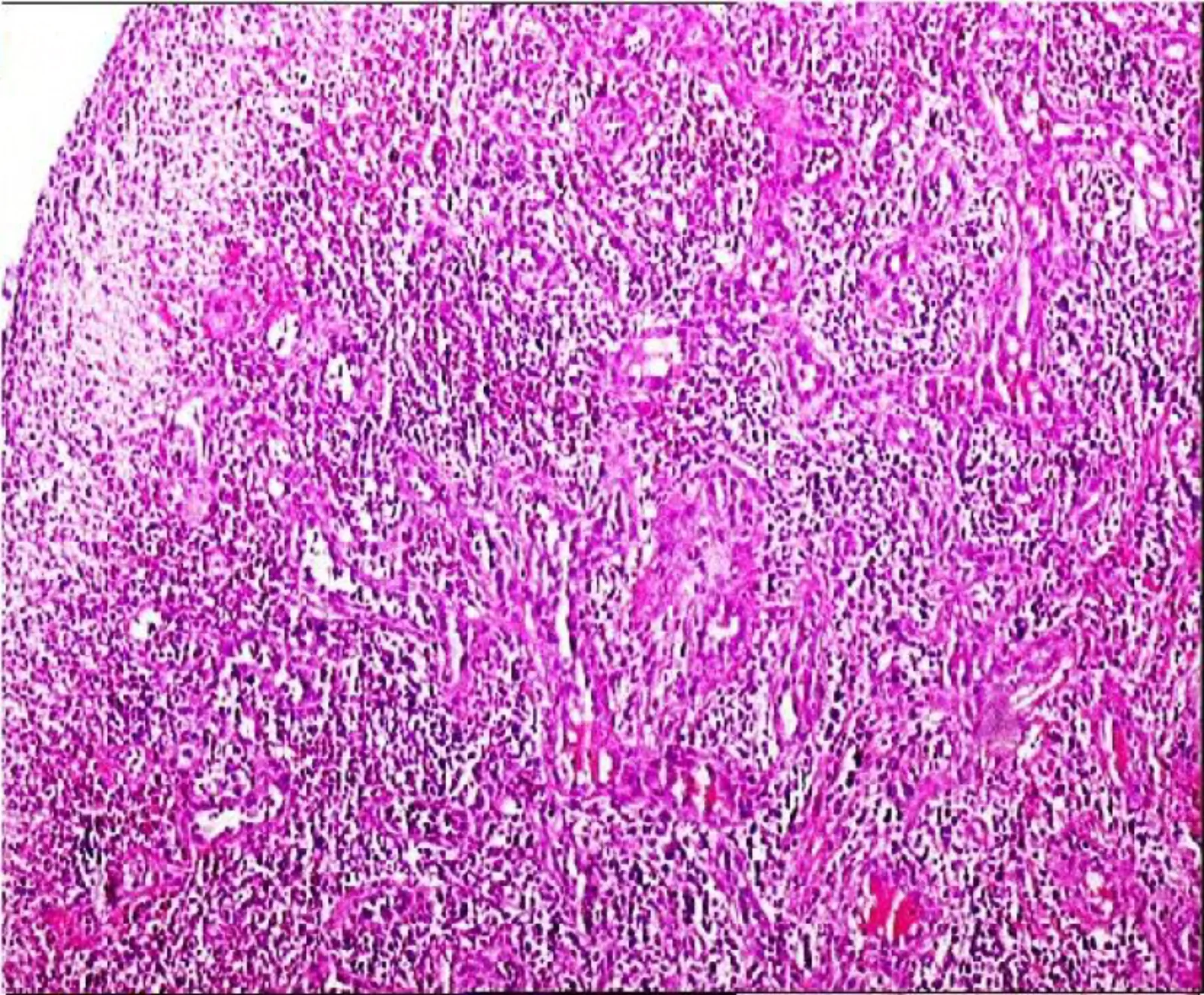
Histopathological image of peri-implant PGCG: histopathological section showing proliferation of fibrohistiocytic cells and numerous multinucleated giant cells separated by a vascularized stroma (H&E ×40). PGCG, peripheral giant cell granuloma.

**Figure 2.**
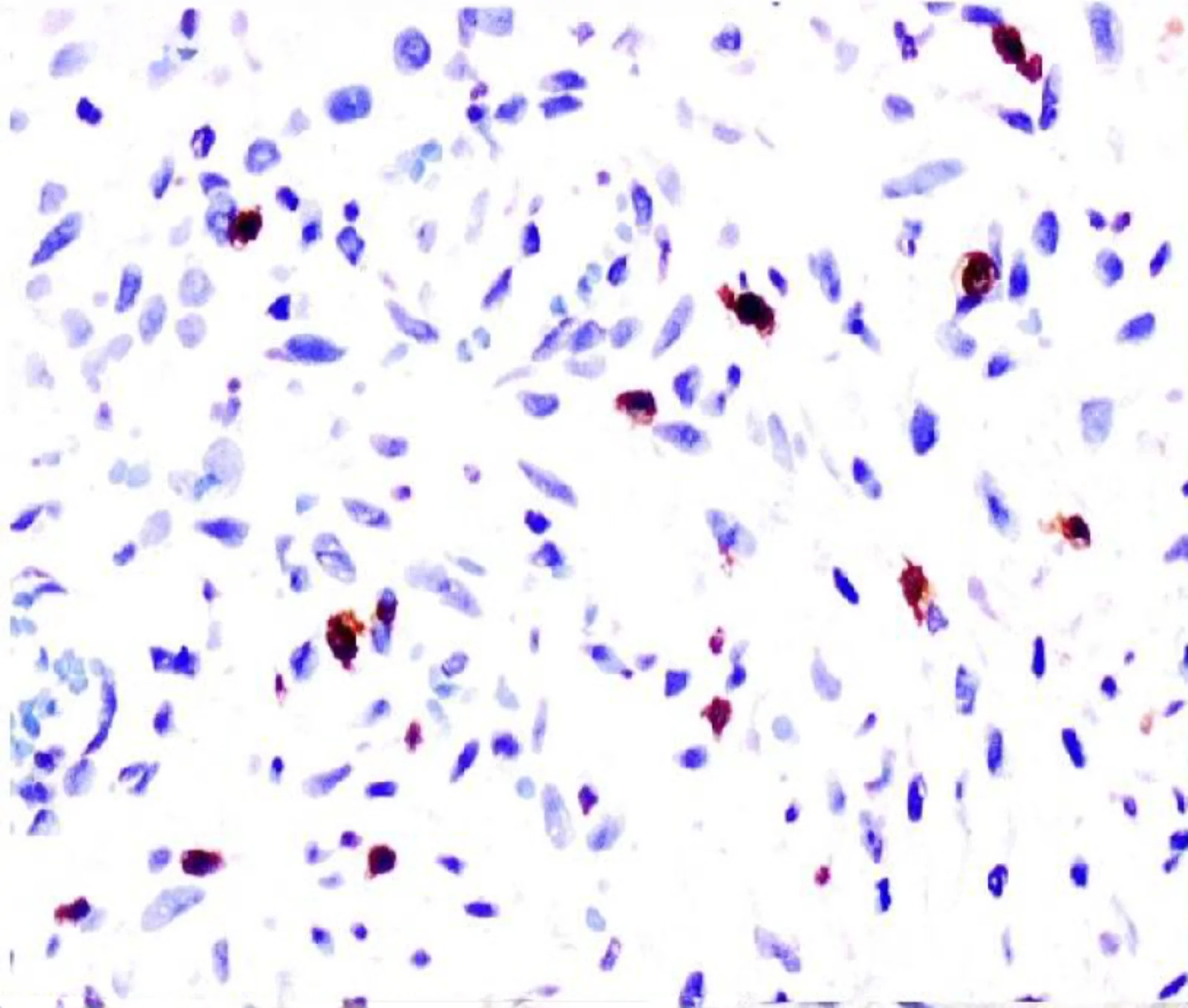
The view of CD3 cells ×200.

**Figure 3.**
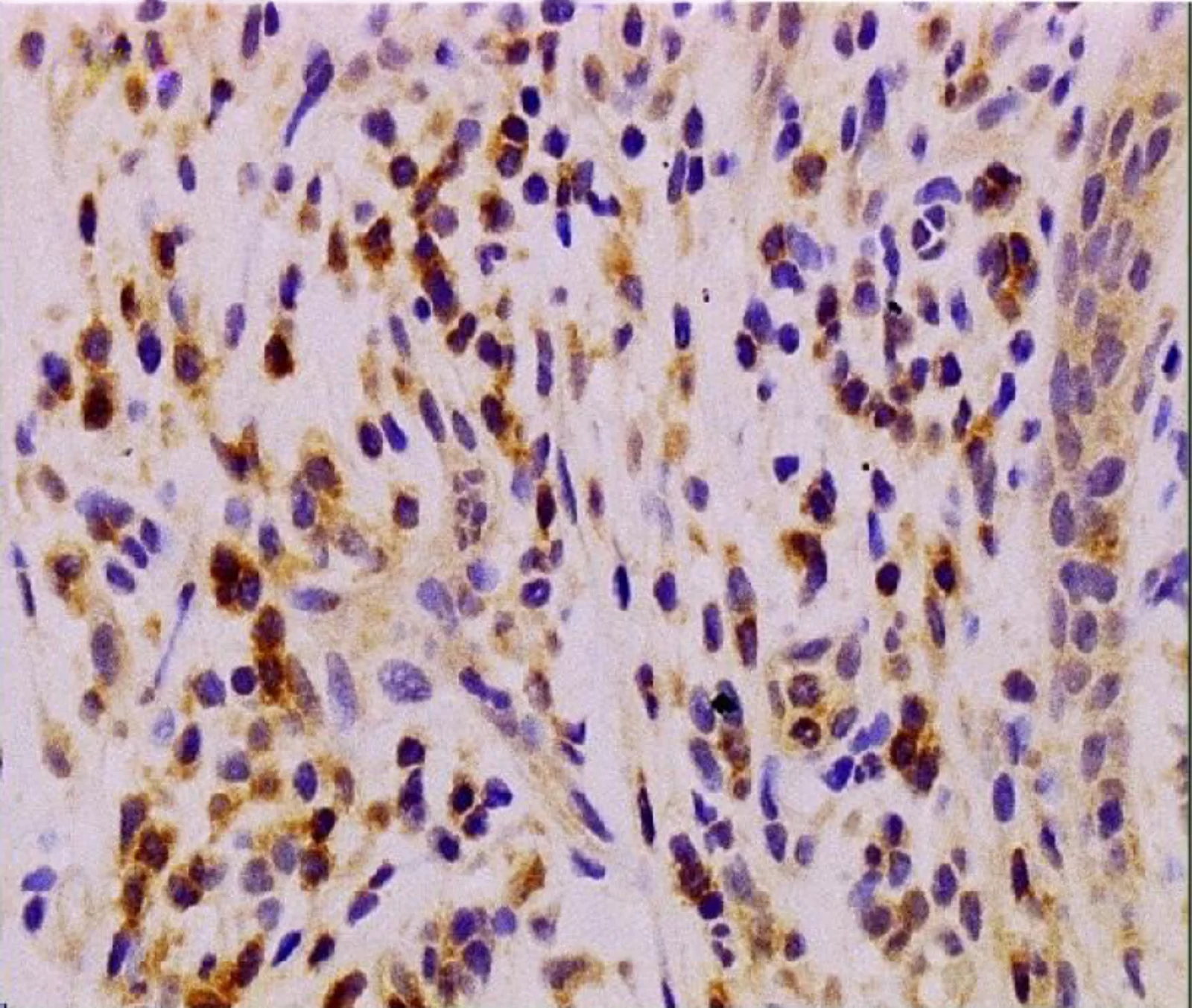
The view of CD4 cells ×200.

**Figure 4.**
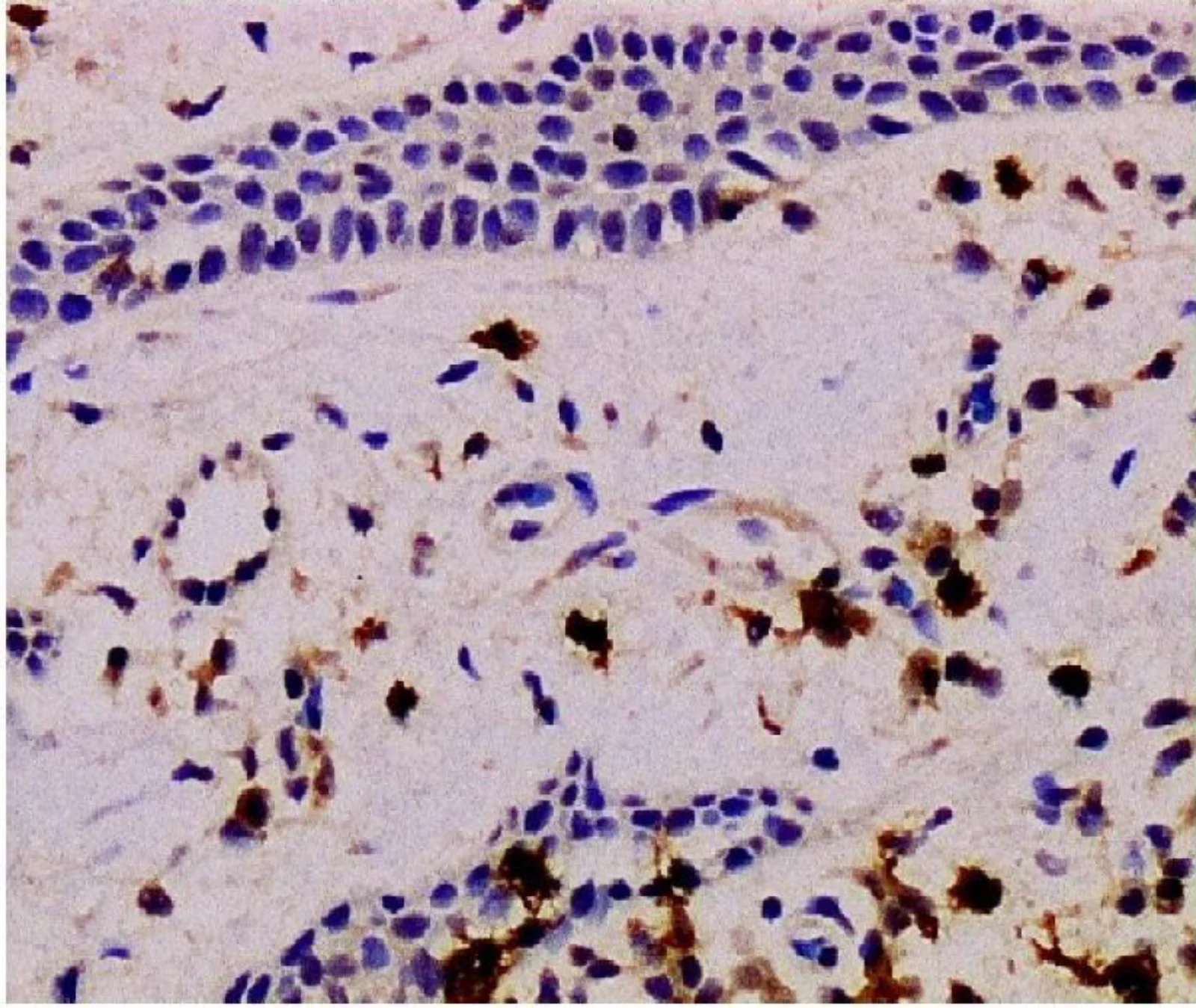
The view of CD8 cells ×200.

**Figure 5.**
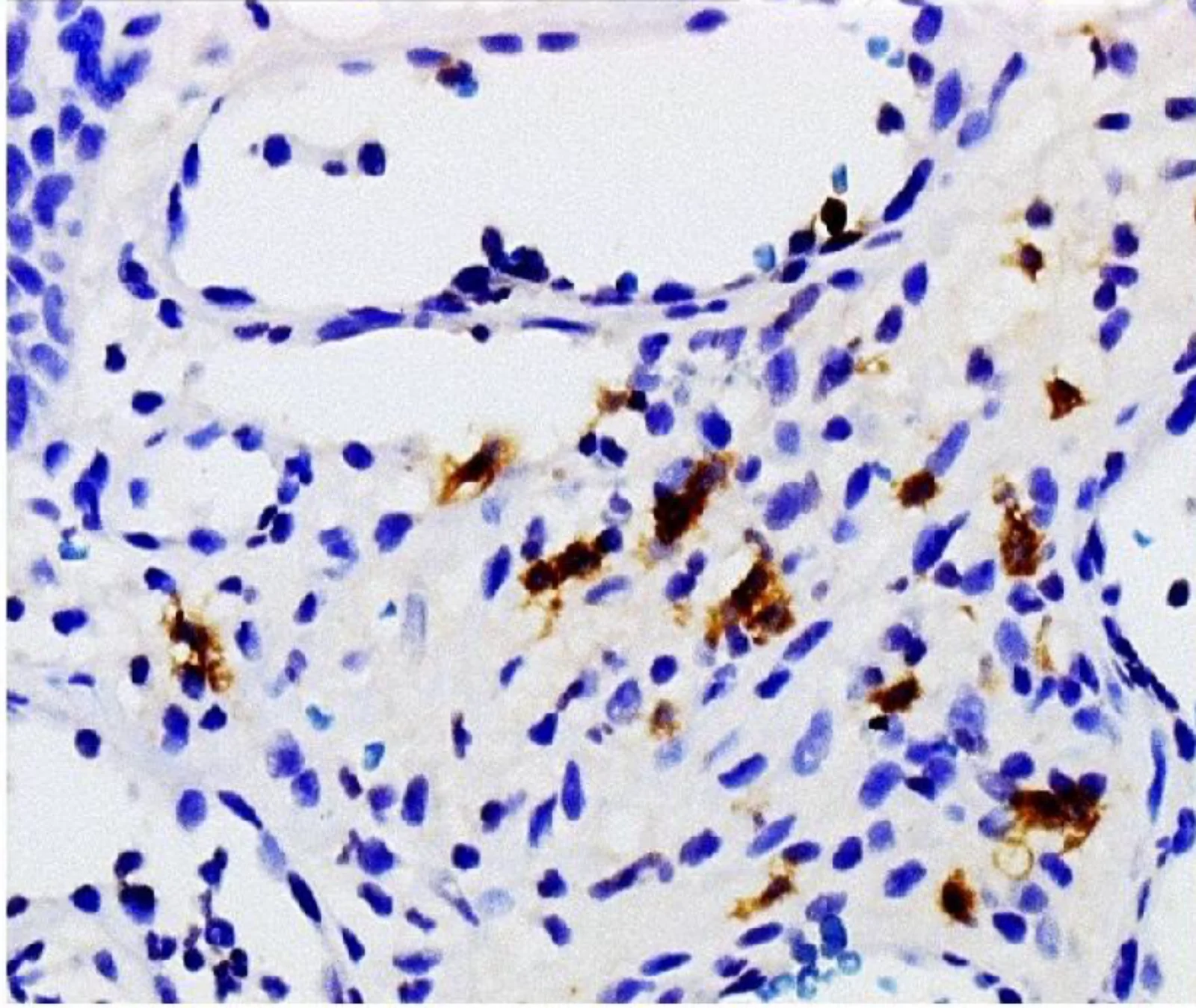
The view of CD20 cells ×200.

**Figure 6.**
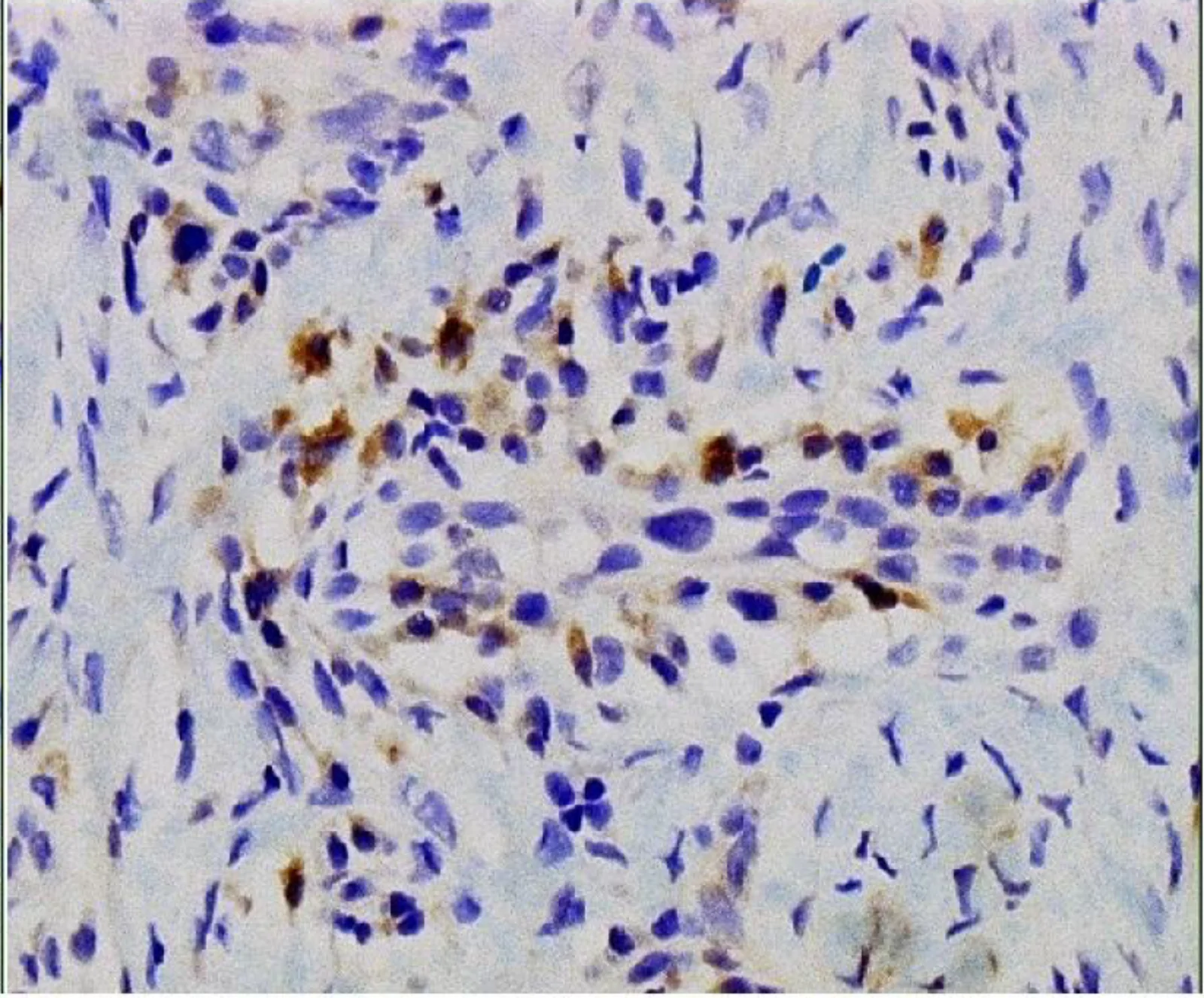
The view of CD79a cells ×200.

**Table 1.** Clinical and Demographic Datas of Peri-implant PGCG

Parameter	Peri-implant PGCG
Age (years)	
Mean	52.6±7.7
Range	39-66
Gender	
Female	18 (64%)
Male	10 (36%)
Site	
Maxilla	9 (32%)
Mandible	19 (68%)
Size	
≤15 mm	19 (68%)
>15 mm	9 (32%)

**Table 2.** Comparison of Mean of the Proportion of Lymphocyte Cells in the 2 Groups

Lymphocyte cells (%)	Test Group (PGCG)	Control Group (Gingiva)	*P*
CD3-pan T cells	15.00 ± 4.71	15.50 ± 4.63	.465
CD4-helper T cell	53.50 ± 7.73	20.00 ± 5.08	.001*
CD8-suppressor T	12.50 ± 4.27	13.50 ± 2.91	.281
CD20-B cells	10.50 ± 4.12	30.00 ± 3.85	.001*
CD79a	12.00 ± 3.46	12.50 ± 4.64	.330

PGCG, peripheral giant cell granuloma.

*Significant differences between groups (Wilcoxon’s signed-rank test).

*P* < .05.

## Data Availability

The data that support the findings of this study are available on request from the corresponding author.
